# Aggressive and Autoaggressive Behaviors in Patients with Autism Spectrum Disorder in Correlation with Middle Cerebral Artery Flow Velocity

**DOI:** 10.3390/jpm14091010

**Published:** 2024-09-22

**Authors:** Maciej Abakumow, Maciej Przybylski, Mariusz Słoma, Olga Markowska, Katarzyna Sowa, Przemysław Jaśkiewicz, Krzysztof Kowalczuk

**Affiliations:** 1 Neures Polska, 04-768 Warszaw, Polandprzemyslaw.jaskiewicz@neures.pl (P.J.); 2 Department of Radiology, Military Institute of Aviation Medicine, 01-755 Warsaw, Poland; 3 Psychological and Pedagogical Counselling Center No. IV, 00-950 Warsaw, Poland; 4 Simulator Research and Aeromedical Training Department, Military Institute of Aviation Medicine, 01-755 Warsaw, Poland

**Keywords:** autism, autism therapy, cerebral artery, ultrasound, aggression, suit

## Abstract

Background/Objectives The purpose of this study was to see whether there is a correlation between the behavior of autism spectrum disorder patients and brain abnormalities based on the velocity of blood flow in the MCA (*middle cerebral artery*). Methods: The use of HAP (*High Altitude Protection*) suits, which are used in aviation, to treat patients with ASD (*autism spectrum disorder*) causes significant changes in their functioning and physiological processes. These changes are not only noted in psychological tests but are observed in cerebral blood flow using transcranial Doppler ultrasound of the MCA. Results The results of this study made it possible to distinguish two groups with different flow velocities, which can be characterized as flows of less than 80 cm/s and flows of more than 80 cm/s. In addition, it was shown that in patients with elevated blood flow velocity, aggressive behaviors account for 86.96%, while self-aggressive behaviors account for 65.2%. On the other hand, in the case of patients with reduced flow velocity, i.e., less than 80 cm/s, the rate of aggressive behavior is 20% and that of self-aggressive behavior is 50%. The experiment showed that after therapy, there is a normalization of blood flow, which increased in the case of patients with a reduced flow rate below 80 cm/s and, in the case of elevated blood velocity after therapy, decreased towards normal levels. Conclusions The observed rate of normalization of flow velocities in the MCA translated into significant changes in the behavior and functioning of patients in the neurotypical direction, which was noticeable in the psychological tests conducted.

## 1. Introduction

The term autism spectrum disorder (ASD) was introduced to the medical world by pediatrician Leo Kanner, who published an article on the subject in the American medical journal *The Nervous Child* in 1943 [1]. However, there are descriptions in the literature that indicate that the problem of “strange behavior in children” existed earlier. As early as the late 18th century, there were descriptions of children affected by disorders whose features did not resemble known disease entities or disorders at the time. Since the beginning of the twentieth century, there have also been scientific descriptions of children’s illnesses that are similar in nature to schizophrenia, yet did not fully meet the criteria for this disease [2].

Autism is treated as a neurodevelopmental disorder, the symptoms of which usually manifest themselves before a child reaches the age of three. Regarding its causes, psychogenic concepts are built on the belief that autism is the result of the appearance of a traumatizing factor (event) during the child’s development [3]. A different view of the problem of etiology is the analysis of factors that can affect the appearance of ASD. These factors are neurological (brain lesions), environmental (prenatal, perinatal, and postnatal factors) and biological (genetic factors, infections, metabolic disorders, immunological factors, neurochemical factors, and hormones and neurotransmitters) [4].

People affected by this disorder manifest deficits in social interaction and verbal and non-verbal communication, as well as displaying disturbed (restricted or stereotypical) behavioral patterns [5].They are most often diagnosed based on two classifications: the DSM (*Diagnostic and Statistical Manual of Mental Disorders*) and ICD (*International Classification of Diseases,*). The ICD-10 classification was introduced in 1992 (WHO—*World Health Organization*, 1992); its newer version from 2022 is known as the WHO classification ICD-11 and it reflects the current understanding of the disorder [6]. Both classifications allow for some flexibility in the diagnostic interpretation of whether, for example, mild social communication problems and slightly repetitive behaviors and interests are sufficiently disabling to make a diagnostic decision [7].

The presented classification and the occurring symptoms force the medical world to search for the causes of ASD and for better diagnostic tools which will help obtain test results that will be completely reproducible and independent. 

In this regard, a different perspective on the related issues is provided through transcranial ultrasound (ultrasound examination), which involves obtaining an image of blood flow in the arteries of the brain using ultrasound waves. The low cost of the examination, the ability to view the structures of the cerebral vessels, the non-invasiveness of the procedure, and the short examination time are important factors in patients with ASD. Not without significance is also the fact that the ultrasound itself has no side or adverse effects, being completely safe for the patient. There is little research on tests of this type present in the available literature [8], and most of the available literature is on adult patients with schizophrenia.

Ultrasound is a mechanical wave phenomenon with a frequency higher than the upper limit of hearing of the human ear, i.e., 16 Khz (the range from 20 to 20,000 Hz produces auditory sensations in humans. Higher frequency ranges, called ultrasound, are inaudible to us) [9]. Its applications involve the generation and detection of ultrasound waves at intensities that do not damage the structure of the medium under examination [10]. Most medical diagnostic applications use ultrasound in the frequency range of 0.3–15 MHz, except for ophthalmic examinations, where frequencies as high as 60 MHz are used [11]. Vascular examinations use frequencies of 1–10 MHZ. At the heart of ultrasound vascular diagnostics are the phenomena of Doppler and echo. The Doppler phenomenon, described in 1842 by Austrian physicist and astronomer Cristian Doppler, is a frequency shift effect associated with motion [12].

The Doppler effect takes advantage of a phenomenon involving a difference in frequency, and thus wavelength, sent out by the wave source and recorded by an observer that moves relative to the wave source. In the case of Doppler ultrasound, the sound source and the observer are located in the same place, that is, in the camera head, while the moving element is the blood in the vessels [13].

The ultrasound wave, incident on blood cells in motion, changes its frequency in proportion to the speed of flow. Echocardiography is a non-invasive method of examining the heart and blood vessels. The electrical–ultrasound transducer is an instrument head containing piezoelectric crystals [14]. The echo from flowing blood cells is encoded with a color, the hue of which depends on the direction of flow. This means that the image of blood flowing toward the head will be color-coded differently than the image of blood flowing away from the head. The image of color-coded structures is superimposed on the image of grayscale structures. This makes it possible to simultaneously observe the image of the flow against the background of the other anatomical structures at the location under study [11].

## 2. Materials and Methods

The collected materials were obtained during a HAP(*High Altitude Protection)* suit therapy program in order to observe the changes occurring during the rehabilitation process [15]. Approval for performing this medical experiment was obtained from the Ethical Committee Evaluating Biomedical Research at the Military Institute of Aviation Medicine in Warsaw (approval numbers 13/2015, 3/2017, and 11/2020).

The purpose of the data collection was to look for medical variables that could indicate the correlation of cerebral blood flow with the functioning of patients with ASD using Doppler ultrasound.

**Figure 1:**
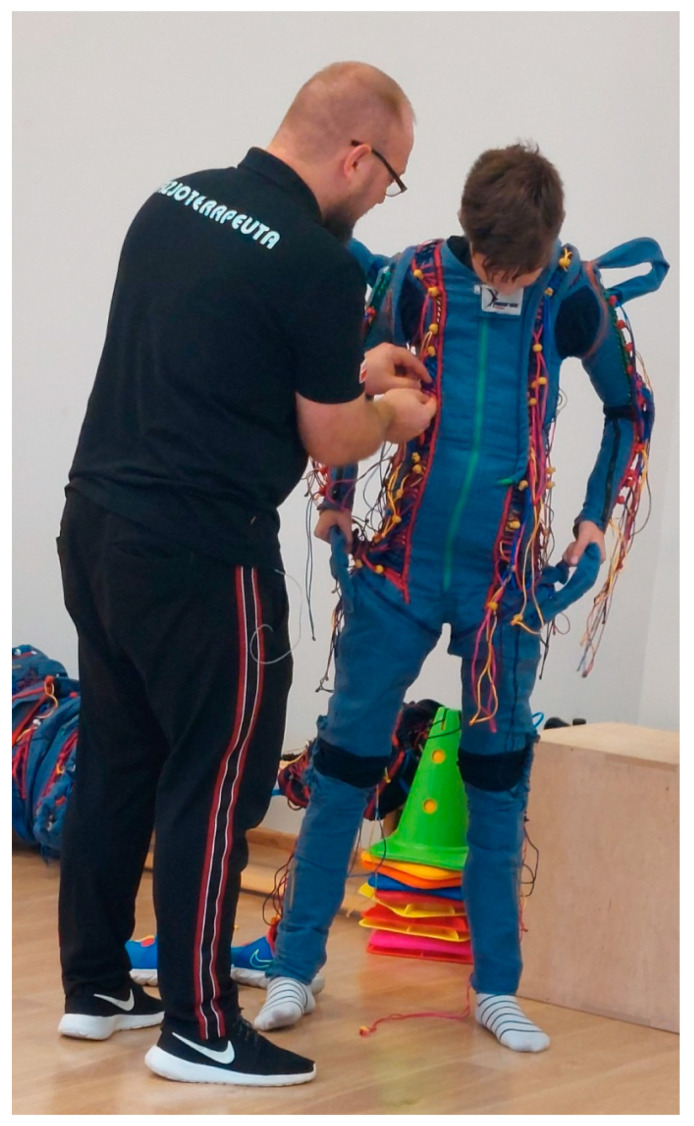
HAP suit—Neures^®^ type, during fitting on a patient. (Own collection of M. Abakumow.).

The methodology used involved ultrasound examinations and psychological testing before and after suit therapy. Before starting the therapy, the suit was selected according to the patient’s height, and standard measurements of blood pressure, heart rate, and saturation were taken. The measurements were taken for patient safety reasons [16,17] This was followed by an exercise phase performed while wearing the suit (Figure 1),which lasted around 45 min. Exercises were performed on a twice-weekly basis for a period of three weeks and then once a week for two weeks and once every two weeks. The total therapy time was 12 weeks.

The exercises used therapeutic methods such as proprioceptive neuromuscular facilitation (PNF) or Bobath neurodevelopmental treatment (NDT) [18,19]. Their main goals included improving eye–hand coordination, balance exercises, breathing exercises, and working in intermediate positions (positions between high positions, such as standing, and low positions, such as crawling).After each session, it was standard practice to measure blood pressure, heart rate, and saturation. After completing a cycle of 10 exercise units, this study was conducted again using psychological tests and ultrasound.

In this research study, we only included patients with official medical certificates stating that they were already diagnosed with ASD.

In order to determine the categories in which patients have the greatest disorders and to determine their level, a standardized Goldstein and Naglieri ASRS *(Autism Spectrum Rating Scales)* psychological test was conducted according to the age of the patients [20,21]. Since there are no direct categories of aggressive or self-aggressive behaviors in the ASRStest, and since these behaviors are the most troublesome to parents or caregivers, the researchers used a proprietary behavioral assessment questionnaire. The questionnaire was completed by the parent or legal guardian in the presence of a psychologist. The questionnaire included behavioral categories and subcategories related to the therapy participants’ disorders, including the following:

*Social behavior*: eye contact, interaction with peers, interaction with adults, functioning in a task situation, functioning in a play situation, thematic play.

*Communication*: responding to commands, using gestures to communicate, communicating one’s needs, dialog speech, narrating on a given topic.

*Rigid patterns*, *activities, and interests*: movement stereotypes (waving hands, spinning), direct echolalia, deferred echolalia, fixations.

*Other common co-occurring disorders*: physical activity, attention, motivation, aggressive behavior, autoaggressive (self-aggressive) behavior.

The questionnaire adopted a seven-point assessment of the patient’s behavior, with negative values marked on a scale from −3 to −1, while positive changes in behavior were marked from 1 to 3. Anadditional value of 0 was introduced for behaviors that did not change and an N value for behaviors that did not occur in the patient. The scale was as follows:

(−3)—great deterioration,(−2)—moderate deterioration, (−1)—slight deterioration, (0)—no change, (1)—slight improvement, (2)—moderate improvement, (3)—great improvement, (N)—the disorder was not present.

Apsychological study was conducted to isolate the subcategories in which the patients’ functioning is disturbed and to their capture aggressive and self-aggressive behavior.

**Figure 2:**
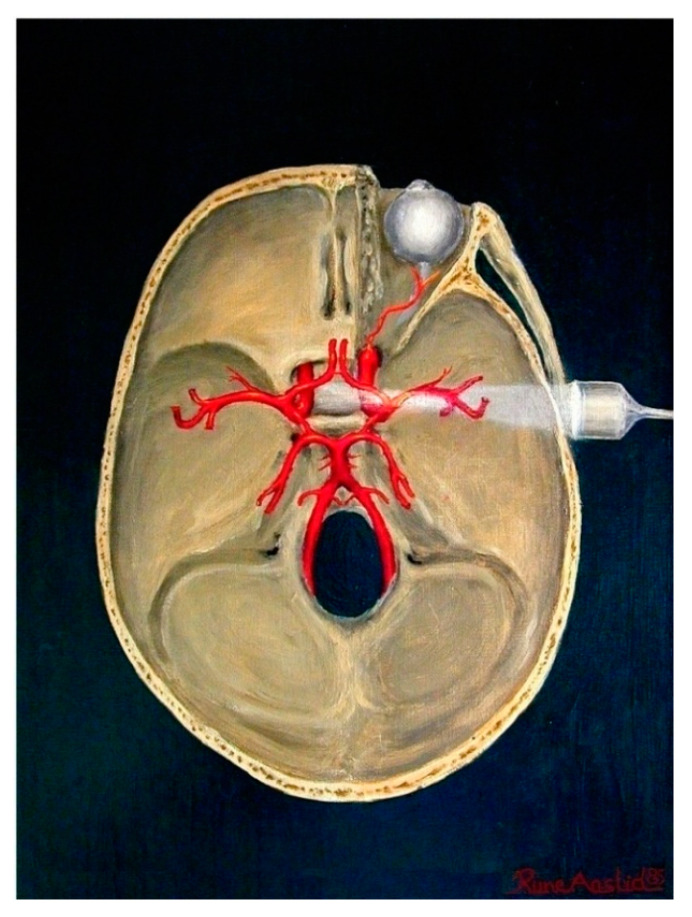
Principle of transcranial MCA Doppler ultrasound [22].

During the research process, Doppler ultrasound of the middle cerebral artery (MCA) was performed (Figure 2). This examination was performed before and after therapy. An instrument manufactured by TOSHIBA (Minato, Tokyo, Japan), model Aplio500, with a sector cardiac head at2–2,5 MHz frequencies, was used for the experiment.

**Figure 3:**
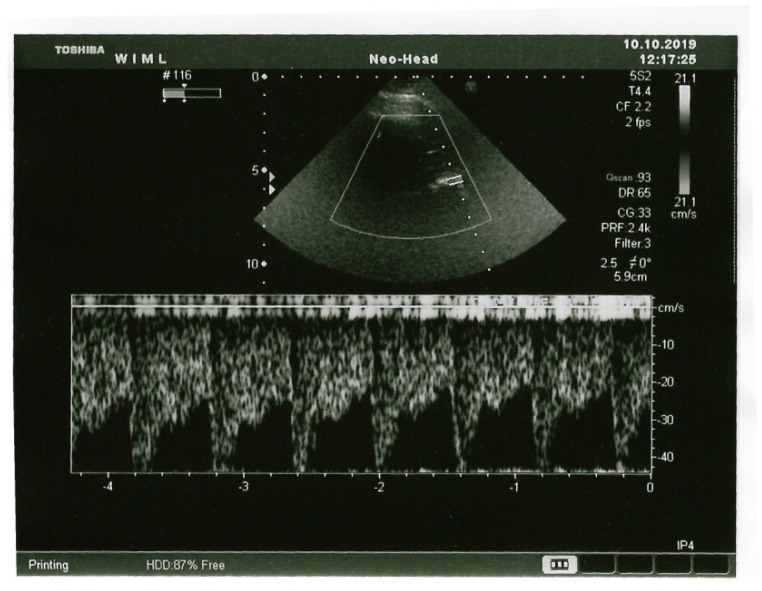
Ultrasound recording of the MCA at the M1/M2 level (M. Abakumow, own collection).

In the case of transcranial examination, the bone layer is a significant barrier to both penetrating and returning ultrasound waves (Figure 3), causing an attenuation ranging from 15 to as much as 60 dB [23]. This attenuation interferes with examination results. Therefore, in order to visualize intracranial structures, acoustic windows are used, which are places within the skull where the bone layer is thinner and allows for the penetration of ultrasound waves. These are the temporal, suboccipital, and transorbital windows [13]. The examination was performed in the supine position after the patient had rested for about 15 min in the presence of a caregiver or parent.

Thirty-three children participated in this study, including four girls and twenty-nineboys between the ages of 5 and 17. The average age was 9.2 years for girls and 8.8 for boys. A cross-section of the study group is shown in Table 1.

## 3. Results

In the conducted study, it was necessary to adopt aso-called norm in the MCA test, which, in the case of healthy patients, is 80 cm/s [24]. With mild vasospasm, the average blood flow velocities increase to 120–159 cm/s, and values above 200 cm/s indicate severe vasospasm and associated cerebral ischemia [25].

**Figure 4:**
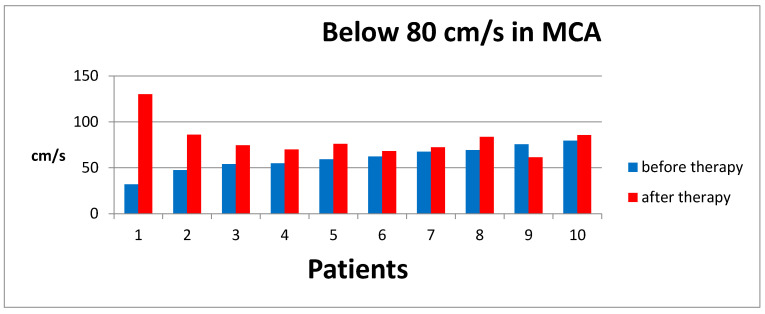
Results of MCA flow velocity on Doppler USG before and after therapy for Group A.

**Figure 5:**
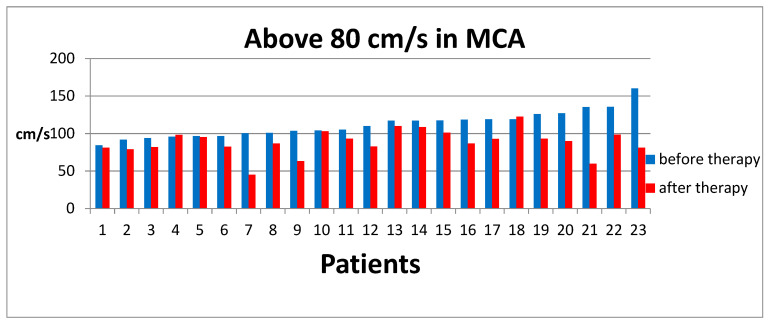
Results of MCA flow velocity on Doppler USG before and after therapy for Group B.

For patients with ASD, the results obtained ranged from 32 cm/s to 160.2 cm/s, with a mean result of 96.25 cm/s, SD ± 28.7. Therefore, the results were divided into values below 80 cm/s (Group A) and values above 80 cm/s (Group B). According to the established methodology, after a series of ten sessions, MCA Doppler ultrasound examinations were performed again. The results of this studyare illustrated in Figure 4 and Figure 5.

The results obtained for Group A, after averaging, were 60.2 cm/s, SD ± 13.33, before therapyand 80.8 cm/s, SD ± 18.05, after the sessions.In Group B, the initial results were 111.9 cm/s, SD ± 17.14, and after the sessions, 88.5 cm/s, SD ±16.59.

The results presented in Table 2 and Table 3 show the changes in the patients’ psychological functioning that were observed by parents of ASD patients during and after therapy grouped by initial flow in the MCA.

Noteworthy is the comparison of the results of changes in the physiological range within the velocity of flow in the MCA. Among patients who experienced a change in mental functioning after therapy, there was also a normalization of flow velocity parameters toward the physiological norm. The average normalization values were as follows:

V_n_= |V_1_−V_0_|

where V_n_ is the absolute value obtained from the difference betweenV_1_ (value of MCA flow velocity after therapy) and V_0_ (value of flow velocity before therapy).When the range was less than 80 cm/s, the average V_n_ was 20.6 cm/s, and for patients whose flow was above 80 cm/s, the average level of normalization of V_n_ was 23.4 cm/s. Another interesting phenomenon in the group of patients with flow rates above 80 cm/s is aggressive behavior (20/23 = 86.96% of patients) and self-aggressive behavior (15/23 = 65.2% of patients).For patients with flow rates below 80 cm/s, the rate of aggressive behavior was2/10 = 20% of patients, and that of self-aggressive behavior was 5/10 = 50% of patients. The results are correlated according to Spearman correlation at r = 0.301, *p*-Value =0.089, which indicates an average correlation; however, in the case of autoaggression, r = 0.401, *p*-Value =0.021, which indicates a strong correlation [26].

A separate issue was the correlation of results between ultrasound flow velocity rates in the MCA. For the studied category of patient communication, the improvement result was 93.4%, with r = 0.477 and *p*-Value = 0.005, which also indicates a strong correlation. Statistical analysis for the category of social behavior, despite 87.87% improvement, showed r = 0.264 for *p*-Value = 0.138, indicating a weak correlation of results.

## 4. Discussion

Issues related to the autism spectrum have been and continue to be the subject of research that addresses many aspects of physiopathology, ranging from changes in DNA structure to socio-educational issues. However, in none of the categories studied is it possible to establish a single cause or a single element whose function is significantly variable, which is a common feature of patients with ASD.

Research has shown that the phenomenon of vasospasm occurs in up to 70% of episodes of subarachnoid hemorrhage and leads to worsening cerebral ischemia and the formation of large neurological defects, which can determine the fate of the patient [27]. An additional danger lies in all clinical conditions in which cerebral flow velocities above 160 cm/s recorded in the MCA are observed [28]. In the case of patients with schizophrenia, increases in blood flow are observed in illness exacerbation states and, at the same time, their decrease and normalization are observed in remission states [29]. There is little research of this type present in the available literature [8], and most of the available literature is on adult patients with schizophrenia.

An increase in flow velocity causes a marked hypoxia of cerebral structures [30]. In the case of patients with ASD, both an increase and a decrease in flow velocity may be associated with hypoxia of cerebral structures [24]. However, it should be remembered that the decrease in flow velocity observed in the MCA is accompanied by a decrease in pressure in the cerebral structures, which results in a decrease in the amount of blood supply to the brain. Since the observed changes involve the MCA, it should be assumed that they occur in all cerebral arteries [31].

The application of HAP suits of the Neures suit type in patients with ASD results in a normalization of blood flow velocities observed in brain structures [15]. The suit not only causes a physiological effect but translates into the patient’s behavior, improves their functioning in society, and reduces impairments in social interaction, impaired development of communication, or a rigid and limited repertoire of behaviors, which are the basis for the diagnosis of ASD in patients [28].The research conducted in this study, although not a clinical trial but a medical experiment, showed that patients who received therapy significantly improved their functioning, which closely correlated with changes in MCA (social behavior improved by 87.87%, communication improved by 93.4%, and aggressive and self-aggressive behaviors decreased by86.96% and 65.2%), while brain flow in the MCA normalized.

The observed changes may indicate hypoxia in brain structures, which causes disruptions in cognitive processes or in the way they are processed in the brain. The lack of proper interpretation of stimuli leads to the emergence of aggressive or autoaggresive states inpatients and of disorders in their social functioning. We suppose that, as in mountain sickness, hypoxia, which causes oxygen deficiency in the body, leads to behaviors of impaired perception, including states of aggression [31]. In the case of ASD patients, a state of hypoxia resulting from inadequate blood flow in brain structures may activate a similar behavioral mechanism.

The restoration of normalization of cerebral blood flow partially or completely eliminates this problem, which may be related to the level of brain damage due to hypoxia or the malnutrition of nerve cells. We know that hypoxia of brain tissue can be caused by an excessively high cerebral blood flow velocity and an excessively low cerebral blood flow velocity [32]. Therefore, it seems reasonable to assume that the normalization of flow velocity causes an improvement in the oxygenation of brain tissue and thus implies a decrease in the severity of symptoms.

## Figures and Tables

**Table 1:** Age distribution of the study group.

AGE	5–6 y.o.	7–8 y.o.	9–10 y.o.	11–12 y.o.	13–14 y.o.	15–16 y.o.	17+ y.o.
Boys	7	10	5	2	2	0	1
Girls	0	0	1	1	1	1	0
Total	7	8	6	3	3	1	1

**Table 2:** Psychological examination after the completion of therapy in patients with blood flow above 80 cm/s.

Behavioral category	<80 cm/s total points	<80 cm/s average points
Social behavior	80	8.0
Communication	79	7.9
Rigid patterns, activities, and interests	23	2.3
Other frequently co-occurring disorders	44	4.4
Total	226	22.6

**Table 3:** Psychological examination after the completion of therapy in patients with blood flow below 80 cm/s.

Behavioral category	>80 cm/s total point	>80 cm/s average points
Social behavior	117	5.1
Communication	92	4
Rigid patterns, activities, and interests	39	1.7
Other frequently co-occurring disorders	67	2.9
Total	315	13.7

## Data Availability

The authors make the raw data underlying the conclusions in this article available upon request.
